# Storage of Mutant Human SOD1 in Non-Neural Cells from the Type-1 Amyotrophic Lateral Sclerosis rat^G93A^ Model Correlated with the Lysosomes’ Dysfunction

**DOI:** 10.3390/biomedicines9091080

**Published:** 2021-08-24

**Authors:** Ilaria Bicchi, Francesco Morena, Chiara Argentati, Laura Rota Nodari, Carla Emiliani, Maurizio Gelati, Angelo L. Vescovi, Sabata Martino

**Affiliations:** 1 Department of Chemistry, Biology and Biotechnology, University of Perugia, Via del Giochetto, 06123 Perugia, Italy; ilabi1983@gmail.com (I.B.); francesco.morena@unipg.it (F.M.); chiara.argentati@unipg.it (C.A.); carla.emiliani@unipg.it (C.E.); 2 IRCCS Casa Sollievo della Sofferenza, Viale Cappuccini 1, 71013 San Giovanni Rotondo, Italy; m.gelati@css-mendel.it (M.G.); vescovia@gmail.com (A.L.V.); 3 Department of Biotechnologies and Biosciences, University of Milano Bicocca, Piazza della Scienza 2, 20126 Milan, Italy; laura.rotanodari@gmail.com

**Keywords:** Hexosaminidase, GALC, LC3, autophagy, mutant SOD1 lysosomal storage, lysosomal storage disorders, bone marrow-mesenchymal stem cells, ALS

## Abstract

Herein, we explored the impact of the lysosome dysfunction during the progression of Amyotrophic Lateral Sclerosis type-1 (ALS1). We conducted the study in non-neural cells, primary fibroblasts (rFFFs), and bone marrow-mesenchymal stem cells (rBM-MSCs), isolated from the animal model rat^G93A^ for ALS1 at two stages of the disease: Pre-symptomatic-stage (ALS1-PreS) and Terminal-stage (ALS1-EndS). We documented the storage of human mutant Superoxide Dismutase 1, SOD1^G93A^ (SOD1*) in the lysosomes of ALS1-rFFFs and ALS1-rBM-MSCs and demonstrated the hallmarks of the disease in non-neural cells as in rat^G93A^-ALS1-tissues. We showed that the SOD1* storage is associated with the altered glycohydrolases and proteases levels in tissues and both cell types from ALS1-PreS to ALS1-EndS. Only in ALS1-rFFFs, the lysosomes lost homeostasis, enlarge drastically, and contribute to the cell metabolic damage. Contrariwise, in ALS1-rBM-MSCs, we found a negligible metabolic dysfunction, which makes these cells’ status similar to WT. We addressed this phenomenon to a safety mechanism perhaps associated with an enhanced lysosomal autophagic activity in ALS1-rBM-MSCs compared to ALS1-rFFFs, in which the lysosomal level of LC3-II/LC3I was comparable to that of WT-rFFFs. We suggested that the autophagic machinery could balance the storage of SOD1* aggregates and the lysosomal enzyme dysfunction even in ALS1-EndS-stem cells.

## 1. Introduction

Amyotrophic lateral sclerosis (ALS, OMIM#105400) is a multigenic/multifactorial progressive neurodegenerative disease with lethality occurring three years after the first symptoms in almost 60% of patients [1]. The high heterogeneity of the disease is under extensive exploration, and currently, several causative genes and mapped loci have been associated with 26 different forms of ALS [1]. All forms have a common terminal degeneration of both upper and lower motor neurons in the central nervous system but differ for driving molecular mechanisms, which in many cases are almost unknown. Many aspects of the pathophysiology of the disease remain still controversial or poorly elucidated even in the most common forms of ALS, such as those caused by mutations in *C9ORF72* (40%), *SOD1* (20%), *FUS* (1–5%), and *TARBDP* (1–5%) genes, respectively for ALS-FTD1, ALS1, ALS6 and ALS10 [2,3,4]. The scenario is worse by the similar onset of ALS with several neurodegenerative diseases (e.g., Alzheimer’s Disease (AD), Huntington Disease (HD), Spinal Muscular Atrophy (SMA), Parkinson’s Disease (PD), Hereditary Spastic Paraplegia (HSP), Fronto-Temporal Disease (FTD), Progressive bulbar palsy (PBP) and some Lysosomal Storage Disorders (LSDs) [4]), that may be a cause of misdiagnoses, especially during the early stages of the disease [4]. Thus, in ALS, AD, PD, HD, and LSDs the formation of aggregates of target proteins and their storage in motor neurons is a common hallmark of the diseases [1,5]. Similarly, abnormal glycosphingolipid metabolism, a feature of SMA, HSP, and LSDs, was also demonstrated in the ALS1 transgenic mice SOD1^G93A^ and cells from patients, and the dysregulation of glucosylceramide content (a sign of LSDs) was also shown in the ALS1 transgenic mice SOD^G86R^ [6]. Moreover, dysfunction of the autophagic-lysosomal network has been documented among above mentioned neurodegenerative diseases. Indeed, whether these dysregulations are a direct causative event of the ALS pathophysiology (e.g., accumulation of undegraded misfolded SOD1 aggregates), or are secondary effects of the progression of the disease, are yet an open question. Therefore, during the last years, the effort has been made on elucidating ALS molecular pathways, also taking advantage of different types of animal models and cell culture models for the different types of the disease [7].

Among the ALS forms, ALS1 occurs in 20% of familial ALS and is highly frequent in sporadic ALS cases [8]. ALS1 is caused by mutations in the superoxide dismutase-1 gene (*SOD1*), located on chromosome 21q22.11, which encodes for the enzyme SOD1 (EC 1.15.1.1) that catalyzes the dismutation of superoxide radicals (O_2_^•−^) to hydrogen peroxide (H_2_O_2_) [9,10,11]. The SOD1 protein is highly expressed, representing 1% of the total cell protein, and is localized mainly in the cytosol and in the inner membrane space of the mitochondria [12]. Moreover, SOD1 has also been demonstrated to be localized in the nucleus, where the enzyme seems to be involved in the genome surveillance processes [13,14]. In both familiar and sporadic ALS1 patients, SOD1 mutations may be the cause either of loss-of-function, with the consequent absence of the protein or its activity, or of gain-of-function, with the production of a toxic misfolded SOD1 protein that assembles into insoluble pathological inclusions and originates the peculiar feature of the human postmortem central nervous system tissues [8,12]. Indeed, the mechanisms of oligomeric SOD1 formation are still deeply exploring, and those leading to the storage of SOD1 aggregates in the cells as well. In this regard, several studies converge on addressing the dysfunction of the cellular degradative pathways (lysosomal, autophagic, and proteasomal systems) as a potential explanation of the accumulation of SOD1 aggregates, but again, the molecular event leading to these network pathways are poorly understood [15].

In this work, we have studied the lysosomes’ activity in the ALS1 animal model, the transgenic rat^G93A^. The study was conducted in tissues, primary fibroblasts (rFFFs), and bone marrow-mesenchymal stem cells (rBM-MSCs) selected as a non-neural cell model of the disease. We selected FFFs as a cell type that often are used as human biobanking of the disease, or for generating induced pluripotent stem cells of ALS1 and related studies [16,17]**,** or to evaluate the mutant SOD1 aggregates formation and the correlation with ALS1 pathology [18,19]. We selected BM-MSCs, due to the numerous researches that evaluate these stem cells in the development of regenerative medicine applications for ALS1 [20,21,22]. Moreover, both FFFs and BM-MSCs also have the advantage to be easily isolated from the tissues, also in ALS1 patients.

Here, we investigated the impact of the mutant SOD1 protein on the lysosome’s functionality by evaluating the expression of a plethora of lysosomal glycohydrolases and proteases, the lysosomal homeostasis and size, the involvement of the autophagic process, and the storage of mutant SOD1 during the progression of ALS1.

## 2. Materials and Methods

### 2.1. Animals

SOD1^G93A^ transgenic Sprague-Dawley male rats, overexpressing the mutated *SOD1^G93A^* human gene, represents a transgenic model of ALS1, were purchased from Taconic Biosciences, Inc. (Hudson, NY, USA; https://www.taconic.com/rat-model/sod1-rat, accessed on 17 March 2021) and bred for the experiments with wild-type Sprague Dawley female rats (Harlan Laboratories, Indianapolis, IN, USA) to obtain and maintain hemizygous transgenic progeny [1,5]. The transgenic offspring were identified by polymerase chain reaction, in which DNA extracted from the tails at P22 was amplified and used to verify the presence of the exogenous human *SOD1* gene [23,24]. All animal care and experimental procedures were carried out according to the current national and international animal ethics guidelines and were approved by the Italian Ministry of Health (authorization number # 286/2013 -B).

Wild type (WT; *n* = 5) and ALS^G93A^ rats at two different stages of the disease: the Pre-symptomatic stage (ALS-PreS; *n* = 5) in which typical ALS symptoms were totally absent at the sacrifice time point (4 months from birth), and the End-stage (ALS-EndS; *n* = 5) in which rats presented total paralysis of hind paws with difficulties to eat in added at the sacrifice time point (6 months from birth). Rats were euthanatized under deep anesthesia and sacrificed at the above indicated time points.

At sacrifice, WT and ALS^G93A^ rats were dissected and several tissues (muscles, liver, spleen, lung, kidney, heart, and brain) were collected and frozen in liquid nitrogen immediately, whereas the paws were collected and kept in sterile physiologic solution (0,9% NaCl) added with 100 U/mL of penicillin-streptomycin (Euroclone S.p.A, Pero (MI), Italy) at 2–8 °C, to avoid contaminations.

Both frozen tissues and fresh paws were used for performing the reported study.

### 2.2. Rat Primary Fibroblasts: Isolation and Culture

Connective tissues, nearer at tibia bone from ALS^G93A^ rats and WT rats has been selected, cut, and transferred, under sterile condition, in 25 cm^2^ tissue culture polystyrene (TCP) flasks in DMEM (Dulbecco’s Modified Eagle Medium, Euroclone S.p.A, Pero (MI), Italy) containing 20% of heat-inactivated fetal bovine serum (FBS), 2 mM L-glutamine, and 100 U/mL of penicillin-streptomycin (Euroclone S.p.A, Pero (MI), Italy) and incubated at 37 °C in a humidified atmosphere with 5% CO_2_. After 7–10 days, primary fibroblasts (rFFFs) spontaneously start to grow out of the tissue and form visible cell colonies. The cells were maintained in the culture medium DMEM contains 10% FBS, 2 mM L-glutamine, and 100 U/mL of penicillin-streptomycin (Euroclone S.p.A, Pero (MI), Italy) (growth culture medium). The medium was refreshed every 3 days. 

### 2.3. Rat Bone Marrow Derived Mesenchymal Stem Cells: Isolation and Culture

The stem cells isolation procedure has been carrying out on the same day for WT animals and ALS1 rat^G93A^, by using the procedure described previously [25,26]. Rat bone marrow-derived mesenchymal stem cells (rBM-MSCs) were isolated from the mononuclear cells obtained by density gradient (Lympholyte; Cedarlane Laboratories Limited, Hornby, ON, Canada) of the bone marrow of the femur and tibiae of two paws of each rat. Mononuclear cells were seeded in a 25 cm^2^ tissue-culture flask in DMEM (Euroclone S.p.A, Pero (MI), Italy) containing 10% of heat-inactivated fetal bovine serum (FBS), 2 mM L-glutamine, and 100 U/mL of penicillin-streptomycin (Euroclone S.p.A, Pero (MI), Italy) (growth culture medium) and incubated at 37 °C in a humidified atmosphere with 5% CO_2_. After 5–7 days of culture, non-adherent cells were discarded, and the fresh medium was added. After 15 days, rBM-MSCs colony became visible. The medium was refreshed every 3 days. 

### 2.4. Cell Proliferation

A growth curve was defined for fibroblasts and rBM-MSCs respectively, to evaluate cells’ growth rate. Parallel curves have been carried out into 48-multiwells plates in duplicate for each cell type. All cells were overnight synchronized removing the FBS in the culture medium. Both rFFFs and rBM-MSCs were plated at a density of 2.5 × 10^2^/well in the growth culture medium and counted every 24 h. For counting, cells were trypsinized and the pellet resuspended in PBS. Cell suspension, added with Trypan Blue solution, was used for the counting procedure by using the Invitrogen^TM^ Counteness^TM^ automated cell counter (Invitrogen™, Grand Island, NY, USA). 

### 2.5. Haematoxylin/Eosin Staining

rFFFs and rBM-MSCs were seeded on glass coverslips at the optimal density of 1 × 10^3^ cells. After fixing with 4% paraformaldehyde for 15 min at room temperature (RT), and some washing with PBS, cells were incubated with 200 μL of Haematoxylin solution (Sigma-Aldrich, St. Louis, MO, USA) for 5 min at RT. After washing with sterile H_2_O, cells were added with 1% of Eosin solution (Sigma-Aldrich, St. Louis, MO, USA) for 3 min at RT and further washed with sterile H_2_O. All procedures were according to the manufacturer’s instructions. Stained cells on coverslips were mounted with Vectashield Antifade Mounting Medium (Vector Laboratories Inc., Burlingame, CA, USA). Image acquisition was performed by using inverted microscopy (Eclipse-TS100, Nikon, Tokyo, Japan) equipped with a digital SIGHT DS-5M-L1 photo camera (Nikon, Tokyo, Japan). 

### 2.6. Multipotential Property of rBM-MSCs from ALS-PreS, ALS1-EndS and WT Rats

The multipotentiality of rBM-MSCs was evaluated testing their differentiation potential toward the osteogenic and adipogenic lineages. In both experiments, 3 × 10^3^ rBM-MSCs were seeded on a 24-wells plate, synchronized overnight with medium FBS-free, and then maintained for 21 days in the differentiation medium. Untreated rBM-MSCs were cultured in the growth medium for the same time as the internal control.

#### 2.6.1. Osteogenic Differentiation

Osteogenic differentiation [26,27] was achieved by culturing rBM-MSCs in mesenchymal stem cell differentiation basal medium-osteogenic (OM) (Lonza Walkersville, Inc., Walkersville, MD, USA) supplemented with the SingleQuots (containing: dexamethasone, ascorbate, L-glutamine, pen/strep, β-glycerophosphate, mesenchymal cell growth supplement (Lonza Walkersville, Inc., Walkersville, MD, USA), for 21 days in a humidified incubator at 37 °C and 5% CO_2_. In all cultures, the medium was changed every 3 days. 

All osteogenic and adipogenic differentiation experiments were performed in triplicate.

Alizarin red staining. The rBM-MSCs osteogenic differentiation was assessed by the analysis of the calcium precipitation through the Alizarin red staining. Briefly, after the osteogenic treatment, the cells were washed with PBS twice, fixed with 4% paraformaldehyde for 15 min at RT, washed in deionized H_2_O, and incubated with 500 μL of 2% Alizarin red solution (Sigma-Aldrich, St. Louis, MO, USA). After, washing with distilled water, the staining was evaluated by the microscopy Eclipse-TS100 (Nikon, Tokyo, Japan) equipped with a digital SIGHT DS-5M-L1 photo camera (Nikon, Tokyo, Japan). 

#### 2.6.2. Adipogenic Differentiation

Adipogenic differentiation [26,27] was performed treating rBM-MSCs with three cycles of induction medium (containing: rh-insulin, mesenchymal cell growth supplement, 3-isobuty-lmethylxanthine, dexamethasone, indomethacin, l-glutamine, penicillin/streptomycin), and maintenance medium (basal medium supplemented with rh-insulin, mesenchymal cell growth supplement, penicillin/streptomycin, l-glutamine) (Lonza Walkersville, Inc., Walkersville, MD, USA), and incubated in a humidified incubator at 37 °C and 5% CO2 for 21 days. In all cultures, the medium was changed every 3 days.

Oil red staining. The adipogenic differentiation was assessed by the analysis of the lipid droplets using the Oil Red O (ORO) staining (BioVision Inc., Milpitas, CA, USA). Briefly, after fixing in 4% paraformaldehyde for 15 min at RT, cultures were washed with PBS, rinsed with 60% isopropanol for 10 min at RT, washed in distilled H_2_O, and stained with 500 μL of ORO solution (ORO 0.3% in isopropanol mixed with H_2_Od (3:2)) for 20 min at RT. Stained cultures were washed twice with distilled H_2_O, and the staining was evaluated by the microscopy (Eclipse-TS100, Nikon, Tokyo, Japan) equipped with a digital SIGHT DS-5M-L1 photo camera (Nikon, Tokyo, Japan). 

### 2.7. Immunofluorescences of rFFFs and rBM-MSCs from ALS-PreS, ALS1-EndS, and WT Rats

Immunofluorescences were performed according to our previous work [28,29,30]. Briefly, cells seeded on glass coverslips at the optimal density of 1 × 10^3^ were fixed with 4% paraformaldehyde, permeabilized, blocked in PBS + 10% FBS, 0.1% Triton X-100 for 1 h at RT, and incubated overnight at 4 °C with the primary antibody, that based on the analyses were: anti-collagen I (COL.1) (1:300, Chemicon International, Temecula, CA, USA) and anti-collagen III (1:300, COL.3 Chemicon International, Temecula, CA, USA); anti-SOD1 (1:400, Santa Cruz Biotechnology, CA, USA). Finally, after incubation with Alexa-Fluor-594 nm or 488 conjugated secondary antibodies (Invitrogen™, Grand Island, NY, USA) for 1 h at RT, coverslips with cells were mounted and nuclei counterstained with Vectashield mounting medium with DAPI (Vector Laboratories Inc., Burlingame, CA, USA). As a negative control, cells on glass coverslip were incubated only with the above indicated conjugated secondary antibodies to verify the absence of autofluorescence or non-specific staining in our samples. 

In some experiments, the F-Actin was stained by incubating the cells with Phalloidin (Alexa-fluor-488 phalloidin, Invitrogen™, Grand Island, NY, USA) for 20 min at RT. 

Images were acquired with fluorescence microscopy (Eclipse-TE2000-S, Nikon, Tokyo, Japan) equipped with the F-ViewII FireWire camera and Cell^f^ software (Soft Imaging System, Olympus, Münster, Germany, version 2.5, Accessed in 2006). The confocal images were acquired with the fluorescence confocal microscopy A1 + Nikon confocal imaging system (Nikon, Tokyo, Japan). 

### 2.8. Acridine Orange Staining of rFFFs and rBM-MSCs from ALS-PreS, ALS1-EndS, and WT Rats

rBM-MSCs and fibroblasts were treated with 5 μg/mL Acridine Orange (Sigma-Aldrich, St. Louis, MO, USA) in the growth culture medium for 15 min at 37 °C. After washing with PBS three times, fixing with 4% paraformaldehyde, further washing with PBS, cultures were mounted with Vectashield mounting medium without DAPI (Vector Laboratories Inc., Burlingame, CA, USA). As a negative control, untreated cells on glass coverslip were subjected to the same procedure. Images were captured with fluorescence microscopy (Eclipse-TE2000-S, Nikon, Tokyo, Japan) equipped with the F-ViewII FireWire camera and Cell^f^ software (Soft Imaging System, Olympus, Münster, Germany, version 2.5, Accessed in 2006).

### 2.9. LysoTracker^®^ Yellow HCK-123 Staining of rFFFs, and rBM-MSCs from ALS-PreS, ALS1-EndS and WT Rats 

The rBM-MSCs and rFFF lysosomes were evaluated by using the vital specific staining LysoTracker^®^ Yellow HCK-123 (Invitrogen™, Grand Island, NY, USA). Cells were treated with 50 nM of the tracer added in the growth culture medium for 30 min at 37 °C, then were immediately fixed with 4% paraformaldehyde, washed twice with PBS, and mounted with Vectashield mounting medium without DAPI (Vector Laboratories Inc., Burlingame, CA, USA). As a negative control, untreated cells on glass coverslip were subjected to the same procedure. Images were captured with fluorescence microscopy F-ViewII FireWire camera and Cell^f^ software (Soft Imaging System, Olympus, Münster, Germany, version 2.5, Accessed in 2006).

### 2.10. FITC-Dextran Staining of rFFFs, and rBM-MSCs from ALS-PreS, ALS1-EndS and WT Rats

FITC-Dextran (Sigma-Aldrich, St. Louis, MO, USA), at the final concentration of 500 μg/mL, was added to the growth culture medium on seeded cells for 30 min at 37 °C. Then, cells were immediately fixed with 4% paraformaldehyde, washed twice with PBS, and mounted with Vectashield mounting medium without DAPI (Vector Laboratories Inc., Burlingame, CA, USA). As a negative control, untreated cells on glass coverslip were subjected to the same procedure. Images were captured with fluorescence microscopy (F-ViewII FireWire camera and Cell^f^ software (Soft Imaging System, Olympus, Münster, Germany, version 2.5, Accessed in 2006).

### 2.11. Images Quantification

Fluorescence intensity quantification of Acridine Orange was performed with a custom-made ImageJ script. For each cell sample (WT, ALS1-PreS, and ALS1-EndS), 50 stained cells were acquired with fluorescence microscopy Eclipse-TE2000-S (Nikon, Tokyo, Japan) and evaluated. Before quantification, some enhancement operations were performed on each image: (i) non-uniform illumination correction, with a Gaussian smoothing filter, was applied [imflatfield function implemented in MatLab (MathWorks, Inc., Natick, MA, USA, version R2019b)], and (ii) background subtraction was done, applying an opening morphological operation, using a disk of 20-pixel diameter [imopen function implemented in Matlab (MathWorks, Inc., Natick, MA, USA, version R2019b)]. Finally, each image was thresholded, cells were masked, and the region properties (integrated density and area) of the masked region of interest (ROI) were calculated for the green and red channels.

A fluorescence intensity projection of the cells was obtained from the relationship:

$$\mathrm{CTCF}=\mathrm{cFID}-\left(\frac{\mathrm{Ac}}{\mathrm{MAB}\times \mathrm{MFB}}\right)$$

where:

CTCF = corrected total cell fluorescence
cFID = cell fluorescence integrated density
Ac = area of masked cell
MAB = mean area of background (mean of five different ROI)
MFB = mean fluorescence of the background (mean of five different ROI)

Finally, the quantitative fluorescence analysis of the Acridine Orange staining was calculated as:

$$\frac{\mathrm{Green}}{\mathrm{Red}}\mathrm{ratio}=\;\overset{n}{\underset{i=1}{\sum }}\frac{\mathrm{CTCF}\;\mathrm{green}}{\mathrm{CTCF}\;\mathrm{red}}$$

For quantification of lysosomes’ size, for each sample, 50 stained cells with Lysotracker and FITC-Dextran, single plane image were acquired with fluorescence microscopy Eclipse-TE2000-S (Nikon, Tokyo, Japan) and evaluated. Both Lysotracker and FITC-Dextran allow us to mark specifically the lysosomes. Before quantification, images were pre-processed (as previously described) and where threshold in FIJI (Fiji Life-Line version, v.2015, National Institutes of Health, Bethesda, MD, USA) with “Auto Local threshold”-> “Phansalkar” [31]. The binary images were used for lysosomes size measurement in FIJI with the “Analyze Particles” tools and “Size (pixel^2^)” parameter set to “50-Infinity. 

### 2.12. Subcellular Localization

The Lysosomal fraction was separated from the cells of ALS1 and WT rats by using our protocol [32,33]. Briefly, 5 × 10^6^ cell pellets were resuspended in 1 mL of 0.25 M sucrose in PBS pH 7.0 at 4 °C and homogenized manually in a Potter-Elvehjem type homogenizer until more than 90% of the cells were broken. For differential centrifugation, the homogenate was centrifuged at 800× *g* for 10 min at 4 °C in a bench centrifuge (5415R Eppendorf, AG, Hamburg, Germany) to sediment the nuclear fraction. The supernatant was then centrifuged at 13,000× *g* for 15 min to sediment the lysosomal (L) fraction and the supernatant from this step was decanted and used as a Cytoplasmic (C) fraction.

### 2.13. Cell and Tissue Extracts

Cells. According to our procedure [34,35], cells were harvested, washed in PBS, resuspended at the concentration of 10^6^ cells/1 mL of 10 mM sodium phosphate buffer, pH 6.0, with 0.1% (*v*/*v*) Nonidet NP40 detergent, and further disrupted by three sonication rounds (30 s each) in ice-cold tubes. Procedures were carried out at 4 °C. 

Tissues. According to previous work [36,37], tissue’s homogenate has been obtained by mechanical homogenization of samples in 10 mM NaPO4 pH 6.0 with 0.1% (*v*/*v*) Nonidet NP-40 detergent (Sigma-Aldrich, Saint Louis, MO, USA) and then subjected to three rounds of sonication, each of 15 s. After 1 h in ice, lysates were centrifuged at 12,000 *g* × 20 min in a refrigerate bench centrifuge (5415R Eppendorf, AG, Hamburg, Germany). Procedures were carried out at 4 °C. Supernatants have been used as tissue extracts. 

### 2.14. Lysosomal Enzyme Activity Assays

Enzyme activities were measured using fluorometric commercially available substrates as described in our previous works [34,35,38,39]. The activity of β-D-N-acetylhexosaminidase (HexA + HexB) and the isoenzyme β-D-N-acetylhexosaminidase A (HexA) were assayed using 3 mM 4-methylumbelliferyl-β-D-N-acetylglucosamine substrate (MUG; Sigma–Aldrich, Saint Louis, MO, USA) and the 3 mM 4-methylumbelliferyl-β-D-N-acetyl-glucosamine-6-sulphate (MUGS, Toronto Research Chemicals,Toronto, ON, Canada) resuspended in 0.1 M citric acid/0.2 M di-sodium phosphate at pH 4.5. The β-Glucuronidase activity was tested with 3 mM 4-metilumbelliferil-β-D-Glucuronide (MUGlu, Sigma–Aldrich, Saint Louis, MO, USA) resuspended in 0.1 M citrate/0.2 M disodium phosphate buffer, pH 4.5. The α-Mannosidase was assayed using 3 mM 4-methylumbelliferyl-α-D-mannopiranoside (MUMan, Sigma–Aldrich, Saint Louis, MO, USA) resuspended in 50 mM sodium acetate/acetic acid pH 4.0. The β-Galactosidase was measured with the 1.5 mM 4-metilumbelliferil-β-D-galactopiranoside (MUGal, Sigma–Aldrich, Saint Louis, MO, USA) prepared in 0.1/0.2 M citrate/phosphate at pH 4.0. The β-Galactocerebrosidase was measured with the 1.5 mM 4-metilumbelliferil-β-D-galactopiranoside (MUGal, Sigma–Aldrich, Saint Louis, MO, USA) in the presence of 11 μM AgNO_3_ resuspended in citrate/phosphate 0.1/0.2 M at pH 4.0. 

All enzymatic assays following the same protocol consisting of 50 μL of sample, 100μL of the substrate, incubation at 37 °C for 30–60 min (depending on the enzyme analyzed), stopping the reaction with ice-cold 0.2 M Glycine/NaOH, pH 10.6. Liberated fluorescent 4-methylumbelliferone was measured in a spectrofluorometer LS-50B Perkin Elmer (λex 360 nm; λem 446 nm). One milliunit of enzyme activity (mU) is defined as the amount of enzyme that hydrolyzes 1 nmol/min of the substrate at 37 °C.

Proteins were measured by the Bradford method using bovine serum albumin as standard [40].

### 2.15. SDS-PAGE and Western Blotting

Protein extracts (30μg each) from ALS1 and WT samples were separated by SDS-PAGE and then subjected to Western blotting and immunodetection with the primary antibodies: anti-SOD1, -Cathepsin S, -Cathepsin D, -Cathepsin B (Santa Cruz Biotechnology, CA, USA) [33,41], -LC3B, (Cell signaling Technology, Danvers, MA, USA), Lamp1 (Sigma Aldrich, St. Louis, MI, USA) and -Actin (Sigma Aldrich, St. Louis, MI, USA), and, with one of the following secondary antibody: Anti-rabbit IgG, HRP-linked Antibody (Cell signaling Technology, Danvers, MA, USA), Anti-mouse IgG, HRP-linked Antibody (Cell signaling Technology, Danvers, MA, USA) Rabbit Anti-Goat IgG Antibody, HRP conjugate (Sigma Aldrich, St. Louis, MI, USA). The ECL^TM^ Detection System (GE Healthcare, Fairfield, CT, USA) was used for the immunostaining procedures. The same blot was re-probed with different antibodies for comparative analyses.

Densitometry analyses were conducted by the FIJI software (FIJI Life-Line version, v.2015, National Institutes of Health, Bethesda, MD, USA). Relative band intensities were normalized to Actin as internal references. Results are expressed as mean ± SD of three independent experiments. 

### 2.16. Statistical Analysis

Western Blotting and Lysosomal Enzymatic Activities Assays studies were performed in triplicate on 3 independent experiments. Data analyses were reported as the mean ± SD (GraphPad 8.0 Software, San Diego, CA, USA). A post-hoc comparison test was performed by the one-way ANOVA and Dunnett’s multiple comparison test with respect to the WT control group (GraphPad 8.0 Software, San Diego, California, USA). *p* ≤ 0.05 was considered statistically significant. 

## 3. Results

### 3.1. Study Model

**Figure 1:**
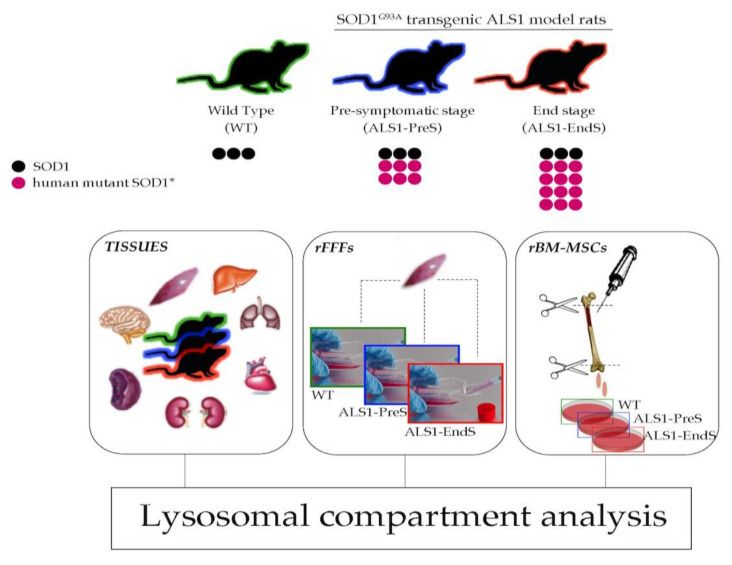
Schematic of the experimental plan for the study of the lysosomal activity in tissues and non-neural cells from transgenic ALS1 rats^G93A^ model. rFFFs: rat Fibroblasts; rBM-MSCs: rat bone marrow-mesenchymal stem cells.

We performed the study using the animal model for ALS1 rat^G93A^ (Sprague-Dawley strand). This transgenic model recapitulates human ALS1 typical hallmarks due to the mutation SOD1^G93A^ in the *SOD1* gene leading to the overexpression of mutant human SOD1^G93A^ (SOD1*) protein and the formation of aggregates among the SOD1* and/or the endogenous naïve SOD1 form. The rat^G93A^ developed the ALS1 disease in 6 months, and, during this period, the level of SOD1* increases drastically and allows to distinguish between two distinct stages of the disease: Pre-symptomatic stage (absence of ALS symptoms; ALS1-PreS), and Terminal stage (exhibition of human typical ALS terminal phenotype; ALS1-EndS) [23]. All experiments were conducted in several tissues and non-neural cells, primary fibroblasts (rFFFs), and bone marrow-mesenchymal stem cells (rBM-MSCs), both isolated from ALS1-PreS and ALS1-EndS rats and were in comparison with parallel analysis in tissues, rFFFs and rBM-MSCs isolated from Sprague-Dawley wild type (WT) rats. In particular, the rat^G93A^-derived tissues were used to set up the expression of SOD1* in the ALS1 model compared to WT, and together with rFFFs and rBM-MSCs, were used to investigate the functionality of the lysosomal compartment in ALS1 (Figure 1). 

### 3.2. Human Mutant SOD1* Is Overexpressed in Tissues, rFFFs, and rBM-MSCs from the ALS1 rat^G93A^ Model 

#### 3.2.1. ALS1-Tissues 

First, we evaluated the SOD1 expression in several tissues from rat^G93A^ and rats WT (Figure S1). We used a primary antibody that reacts with both human and rat SOD1. As expected [23], all rat^G93A^ tissues have the overexpression of SOD1* protein (Figure S1A,B). Of note, we found the same expression pattern of the SOD1 protein consisting of one band in all WT tissues, corresponding to the naïve SOD1, and two bands corresponding to the naïve SOD1 and human mutated SOD1* protein in all ALS1-PreS and ALS1-EndS rat-derived tissues (Figure S1A). The expression levels of the SOD1 and SOD1* in both ALS1 stages differ among tissues: while in the brain, muscle, heart the SOD1* protein was above ~3-fold increase to SOD1 (Figure S1A,B), in the liver, lung, kidney, and spleen the increase was ~1.5-fold. We also observed a decrease of the naïve SOD1 band in kidney and spleen with the onset of symptoms respect to WT counterparts (Figure S1A,B).

#### 3.2.2. ALS1-rFFFs Model 

**Figure 2:**
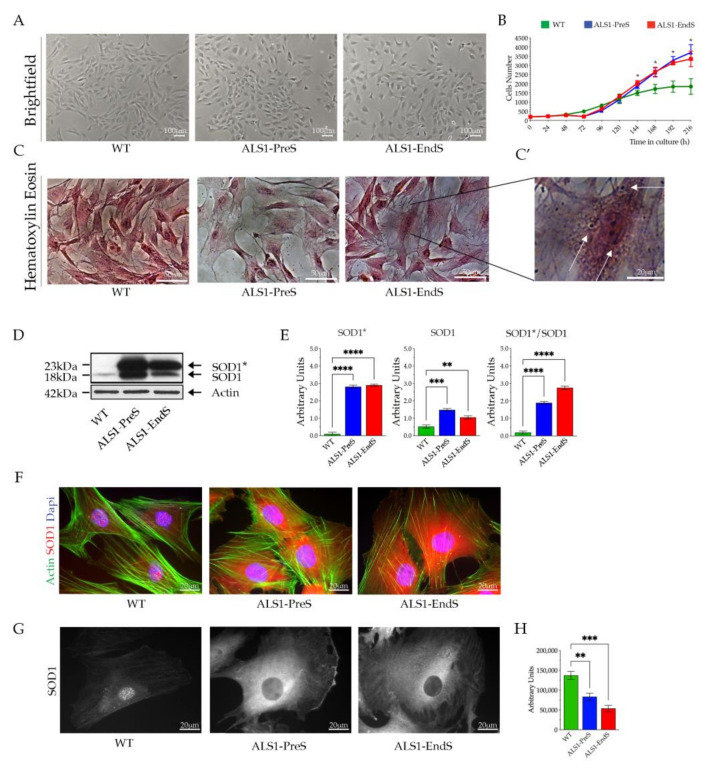
Expression of SOD1* in rFFFs. (**A**) Representative brightfield images of WT, ALS1-PreS, and ALS1-EndS fibroblasts. Scale bar = 100 mm. (**B**) Growth curve of diseased and WT rFFFs. Each point represents the mean of three technical replicates; * *p* < 0.05. (**C**) Representative images of Hematoxylin/Eosin (Scale bar = 50 mm) and (**C’**) high magnification of the ALS1-EndS cells. Scale bar = 20 mm. (**D**) Representative Western blotting analysis of naïve SOD1 and human mutant SOD1* in ALS1 and WT rFFFs. (**E**) Densitometric analysis of the SOD1*, SOD1, and SOD1*/SOD1 ratio. Results are reported as mean ± SD. ** *p* < 0.01, *** *p* < 0.001, **** *p* < 0.0001. (**F**) Representative immunofluorescences of SOD1 expression (anti-SOD1; red) and F-Actin staining (Alexa-fluor-488 Phalloidin, green); Nuclei (4′,6-diamidino-2-phenylindole, DAPI, blue), Scale bar = 20 µm. (**G**) Representative confocal images of immunofluorescence of SOD1 (anti-SOD1) in the rFFFs cells. Scale bar = 20 µm and (**H**) nuclear SOD1 fluorescence quantification.

ALS1-rFFFs, shown the canonical fibroblasts morphology, grown in adhesion to the culture flasks (Figure 2A), and expressed Collagen Type I and Type III as WT rFFFs (Figure S2, Supplementary File). 

ALS1-PreS and ALS1-EndS rFFFs showed a similar growth curve that resulted in a high increase with respect to WT cells (Figure 2B). These data were confirmed by the measure of the Doubling Time (DT), using the Doubling Time tool (https://www.doubling-time.com, accessed on 21 April 2021), which was 41.4 h in WT, 37.2 h in Pre-symptomatic, and 34.7 h in End-stage cells (Figure 2B). Of note, the Haematoxylin/Eosin staining shed light on the presence of acidophil granules in the ALS1-EndS rFFFs and their absence in WT and ALS1-PreS cells (Figure 2C,C’, magnification of representative images, white arrows). 

The Western blotting analysis demonstrated a significant overexpression of SOD1* in rFFFs ALS1-models and its absence in WT cells (Figure 2D,E). As above-described for the ALS1 tissues, we detected one band corresponding to naïve SOD1 in WT rFFFs and, two bands corresponding to naïve SOD1 and SOD1* forms in pre-symptomatic and terminal-stage cells. The densitometric analysis confirmed a 2/3-fold increase of the expression of mutated SOD1* in ALS1-rFFFs compared to the SOD1 band and foremost the increase of human SOD1* protein with the progression of the disease (Figure 2D,E). The immunofluorescence analysis confirmed the highest overexpression of SOD1* in ALS1-cells and revealed a widespread distribution, almost cytoplasmatic, of SOD1* in ALS1-rFFFs, and the cytoplasmatic and nuclear localization of SOD1 in WT cells (Figure 2F). A reduced level of SOD1 in the nuclei with the progression of the disease was also shown by confocal microscopy and related fluorescence quantification (Figure 2G,H).

#### 3.2.3. ALS1-rBM-MSCs 

**Figure 3:**
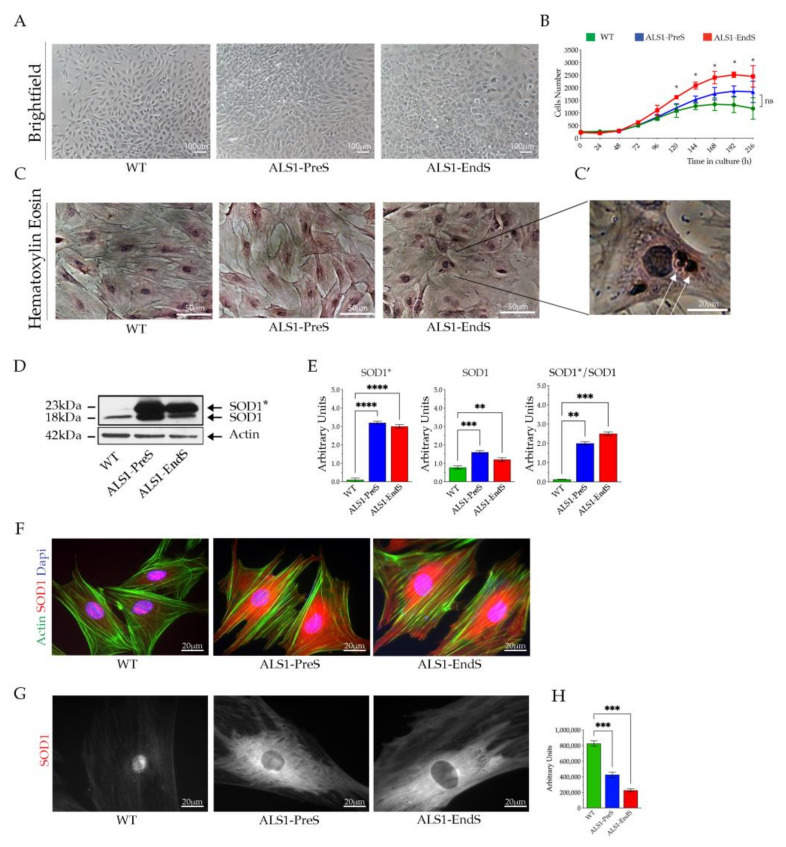
Expression of SOD1* in rBM-MSCs. (**A**) Representative brightfield images of WT, ALS1-PreS, and ALS-EndS stem cells. Scale bar = 100 μm. (**B**) Growth curve of diseased and WT rBM-MSCs. Each point represents the mean of three technical replicates; * *p* < 0.05. (**C**) Representative images of Hematoxylin/Eosin (Scale bar = 50 μm) and (**C’**) high magnification of the ALS1-EndS stem cells. Scale bar = 20 μm. (**D**) Representative Western blotting analysis of naïve SOD1 and human mutant SOD1* in ALS1 and WT rBM-MSCs. (**E**) Densitometric analysis of the SOD1*, SOD1, and SOD1*/SOD1 ratio. (**F**) Representative immunofluorescences of SOD1 expression (anti-SOD1; red) and F-Actin staining (Alexa-fluor-488 Phalloidin, green); Nuclei (4′,6-diamidino-2-phenylindole, DAPI, blue), Scale bar = 20 μm. (**G**) Representative confocal images of immunofluorescence of SOD1 (anti-SOD1) in the rBM-MSCs cells. Scale bar = 20 μm, and (**H**) nuclear SOD1 fluorescence quantification. Results are reported as mean ± SD. ** *p* < 0.01, *** *p* < 0.001, **** *p* < 0.0001.

WT, ALS1-PreS, and ALS1-EndS rBM-MSCs have a typical mesenchymal fibroblasts-like morphology and grow in adhesion to the culture flasks (Figure 3A), but differ for the proliferation rate (Figure 3B). This envisaged by the higher values of both ALS1-PreS and ALS1-EndS stem cell growth curves when compared to the WT, as well as by the DT that was 36.4 h in ALS1-EndS, 42.7 in ALS1-PreS, and 46.7 in WT stem cells, suggesting an increase of the cell proliferation rate with the progression of the disease (Figure 3B). The rBM-MSCs Haematoxylin/Eosin staining confirmed the mesenchymal stem cell shape but also revealed the presence of acidophil granules in the ALS1-EndS stem cells that were absent in ALS1-PreS and WT stem cells (Figure 3C,C’, magnification of the representative image, white arrows).

No differences were observed in the stem cell multipotent property between rBM-MSCs from rat ALS1 model and WT counterpart, as demonstrated by their differentiation toward osteogenic and adipogenic lineages (Figure S3A,B, Supplementary File). 

The expression of SOD1* was found in ALS1-rBM-MSCs, while it was absent in WT stem cells, as shown by Western blotting analysis (Figure 3D,E). Yet, we detected one band corresponding to the naïve SOD1 in WT rBM-MSCs, and two bands corresponding to the naïve SOD1 and mutated SOD1* proteins in ALS1-PreS and ALS1-EndS stem cells. Again, the SOD1* level was almost 2/3-fold higher than the SOD1 band and increased with the progression of the disease (Figure 3D,E). As above-described in rFFFs, the SOD1* was almost spread in the stem cell cytoplasm, whereas the naïve SOD1 was both cytoplasmatic and nuclear (Figure 3F,G), as showed by the reduced signal in the nuclei of ALS1 stem cells (Figure 3H).

Together these results demonstrated that ALS1-rFFFs and rBM-MSCs exhibited the hallmarks of ALS1 as in the rat^G93A^ ALS1 tissues and could be suitable models for molecular investigation of the pathophysiology of the disease *in-vitro*.

### 3.3. The Activity of Lysosomal Glycohydrolases and the Expression of Some Relevant Proteases Are Altered in Tissues, rFFFs, and rBM-MSCs from the ALS1 rat^G93A^ Model

**Figure 4:**
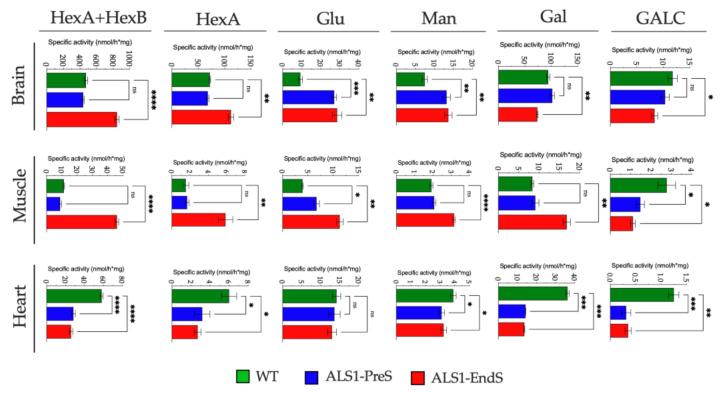
Activity of lysosomal glycohydrolases in tissues from ALS1 and WT rats. Levels of enzymes were measured by using specific fluorogenic substrates (see Section 2.14 for details). Results were expressed as the mean ± SD of three independent experiments, each in triplicates. * *p* < 0.05, ** *p* < 0.01, *** *p* < 0.001, **** *p* < 0.0001.

**Figure 5:**
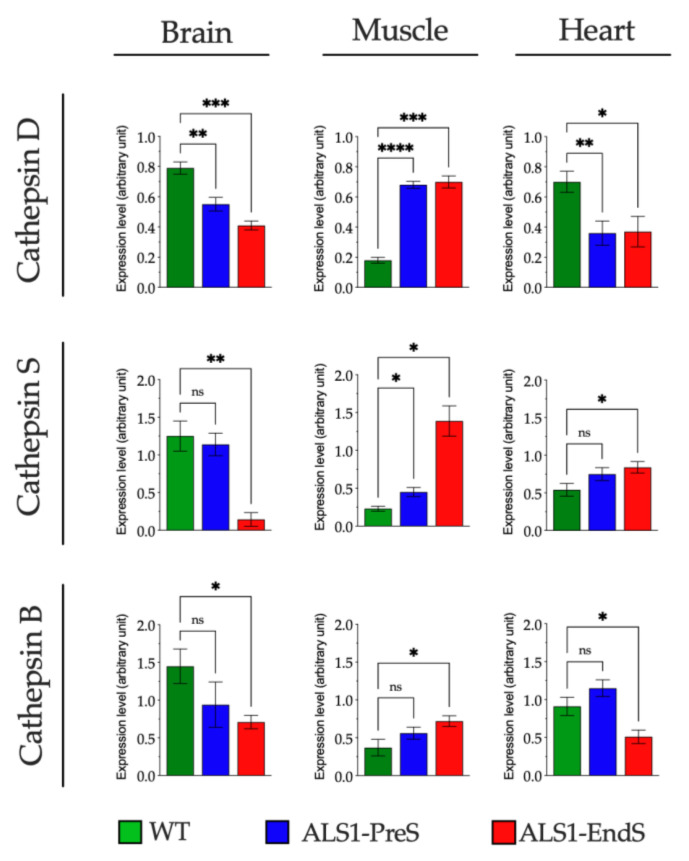
Expression of lysosomal proteases in tissues from ALS1 and WT rats. Levels of proteases were determined by Western blotting (see also Figure S4A, Supplementary File). Shown are the densitometry analyses of all experiments. Results were expressed as the mean ± SD of three independent experiments, each in triplicates. ns, not significant, * *p* < 0.05, ** *p* < 0.01, *** *p* < 0.001, **** *p* < 0.0001.

**Figure 6:**
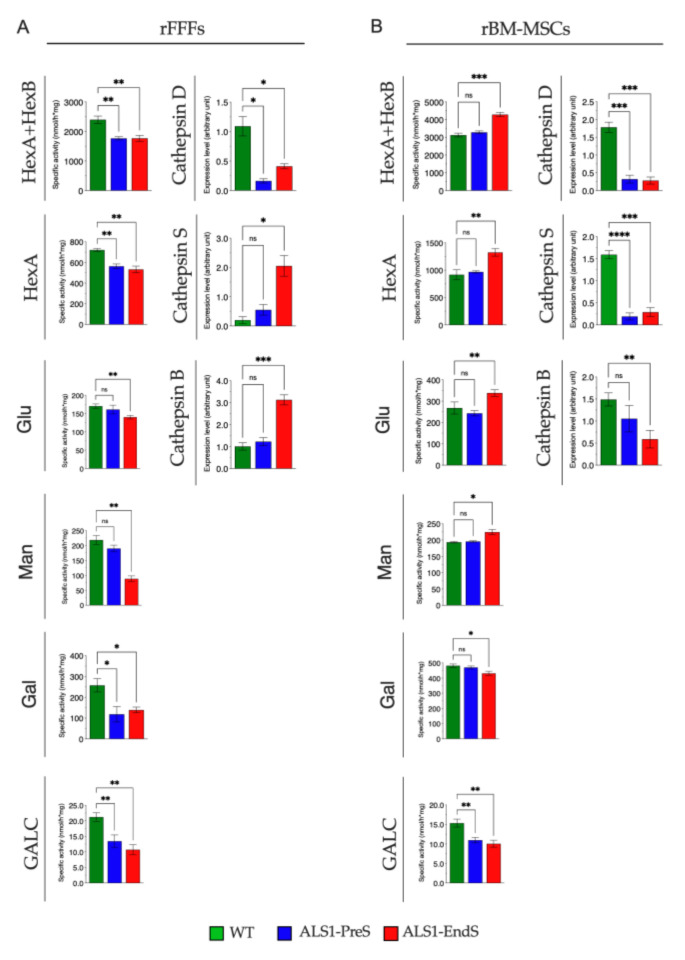
The activity of lysosomal glycohydrolases and expression levels of proteases in rFFFs (**A**) and rBM-MSCs (**B**) from ALS1 and WT rats. Levels of glycohydrolases were measured by using specific fluorogenic substrates (see Section 2.14 for details). Levels of proteases were determined by Western blotting (see also Figure S4B,C in Supplementary File). Shown are the densitometry analyses of all experiments. Results are expressed as the mean ± SD of three independent experiments, each in triplicates. ns, not significant, * *p* < 0.05, ** *p* < 0.01, *** *p* < 0.001, **** *p* < 0.0001.

To investigate the lysosomal compartment functionality in ALS1, we first analyzed the activity of a plethora of lysosomal glycohydrolases (β-Hexosaminidase isoenzymes (HexB, HexA), β-Glucuronidase (Glu), α-Mannosidase (Man), β-Galactosidase (Gal), and Galactocerebrosidase (GALC), and the expression of the lysosomal proteases Cathepsin D (CatD), Cathepsin S (CatS), Cathepsin B (CatB) in the brain, muscle and heart tissues (selected due to the highest overexpression of SOD1*), rFFFs, and rBM-MSCs from ALS1 and WT rats (Figure 4, Figure 5 and Figure 6). 

#### 3.3.1. Lysosomal Glycohydrolases and Proteases in ALS1 Tissues

The Hexosaminidase activity was evaluated through two substrates: one that is hydrolyzed by both HexB and HexA isoenzymes (from now HexB + HexA) and one which is hydrolyzed only by the HexA isoenzyme [35,42]. 

We found a significant increase of Hex activity in the ALS1-EndS brain tissue (HexB + HexA: 79%, HexA: 55%) and muscle tissue (HexB + HexA: >100%, HexA: >100%) when compared to the levels measured in WT and ALS1-PreS counterparts that were comparable (Figure 4), whereas, an opposite reduced trend of Hex activity was measured in the ALS1-PreS and ALS1-EndS heart tissue (HexB + HexA: ~50%; HexA: ~50%) compared to WT (Figure 4).

A significant change of Glu activity was observed in the brain tissue (ALS1-PreS >100% to WT; ALS1-EndS >100% to WT), and in the muscle tissue (ALS1-PreS > 70% to WT; ALS1-EndS > 60% to WT), whereas no variations were observed in both ALS1-PreS and ALS1-EndS heart tissue with respect to the WT (Figure 4). 

We found a similar increase of Man activity in ALS1-PreS and ALS1-EndS brain tissue (77% and 83% to WT), whereas we measured a 65% increase only in ALS1-EndS muscle tissue compared to ALS1-PreS and WT, and a 20% significant reduction in the heart tissue at both stage of disease compared to WT (Figure 4). 

Altered levels of the Gal activity in ALS1 brain, muscle, and heart were also observed, although in a varied range. Thus, Gal activity was reduced in ALS1-EndS brain (20% to ALS1-PreS and WT) and in both ALS1-PreS and ALS1-EndS heart tissues (70% to WT), while it was increased in ALS1-EndS muscle (>100% to ALS1-PreS and WT) (Figure 4). 

The levels of GALC activity were slightly reduced in the ALS1-EndS brain (29% to ALS1-PreS and WT), whereas the enzymatic level was highly decreased in both ALS1-PreS and ALS1-EndS muscle (~50%) and heart (75%) tissues with respect to WT tissues (Figure 4).

The analysis of some lysosomal proteases revealed a different expression level between ALS1 and WT brain, muscle, and heart tissues (Figure 5; Figure S4A in Supplementary File).

The CatD expression decrease in ALS1-PreS (30%) and in ALS1-EndS (48%) brain tissue compared to WT, and in heart tissue at both ALS1 stages (50%) with respect to WT tissues, while CatD levels increased with the progression of the disease (ALS1-PreS > 100%; ALS1-EndS > 100%) in the muscle tissue compared to WT counterparts (Figure 5; Figure S4A in Supplementary File). 

The CatS levels were significantly reduced in ALS1-EndS (88%) versus ALS1-PreS and WT brain tissue counterparts, whereas the protease had a significant increase in muscle ALS1-PreS (95% to WT) and ALS1-EndS (>100% to WT), and in heart tissue at the terminal stage of the disease (55% to ALS1-PreS and WT) (Figure 5; Figure S4A in Supplementary File). 

Finally, we found a reduced expression of CatB in the ALS1-EndS brain and heart (52% and 44%, respectively) with respect to WT counterparts, which were comparable with the levels of ALS-PreS, instead of CatB levels that increased with the progression of the disease (ALS1-EndS 95% to WT) in muscle tissue (Figure 5; Figure S4A in Supplementary File).

#### 3.3.2. Lysosomal Glycohydrolases and Proteases in ALS1-rFFFs- and ALS1-rBM-MSCs

ALS1-rFFFs. Both levels of glycohydrolases and proteases resulted altered with a varied range in pathological compared to WT rFFFs (Figure 6A). 

In detail, HexA + HexB, Hex A, Gal, and GALC activity decreased (26%, 20%, ~50%, ~50% respectively) in both ALS1-PreS and ALS1-EndS FFFs, while Glu and Man activity were reduced, 20% and 60% respectively, only in the ALS1-EndS FFFs with respect to the ALS1-PreS and WT counterparts (Figure 6A). 

Finally, while the level of CatD was significantly decreased in ALS1-PreS and ALS1-EndS (85% and 62%, respectively), CatS and CatB were unchanged in the Pre-symptomatic stage and increased (>100%) in ALS1 End-stage-derived cells when compared with the WT counterpart (Figure 6A; Figure S4B, Supplementary File).

ALS1-rBM-MSCs. Yet, the measures of glycohydrolases activity and protease levels confirmed the different expression in ALS1-rBM-MSCs during the progression of the disease with respect to WT (Figure 6B). 

We found an almost varied increase of HexA + HexB (40%), HexA (44%), Glu (26%), Man (16%), and a decrease of Gal (28%) activity in ALS1-EndS stem cells compared to ALS1-PreS rBM-MSCs which have a similar expression to WT, and a decrease of GalC activity in stem cells at both stage of the disease (28% and 27%, respectively) (Figure 6B). 

In rBM-MSCs, the levels of the CatD, and CatS, were significantly decreased (>100%) at the ALS-PreS and the End-stage of the disease with respect to WT counterpart (Figure 6B), whereas, CatB decreased with the progression of the disease (ALS1-EndS >100% to WT) (Figure 6B; Figure S4C, Supplementary File).

The overall data emphasized that the levels of lysosomal enzymes were altered in ALS1 brain and other tissues and non-neural cell types analyzed with respect to the WT counterparts.

### 3.4. Lysosomes’ Homeostasis and Size Are Highly Altered in ALS1-rFFFs Compared to ALS1-rBM-MSCs In Vitro Models

**Figure 7:**
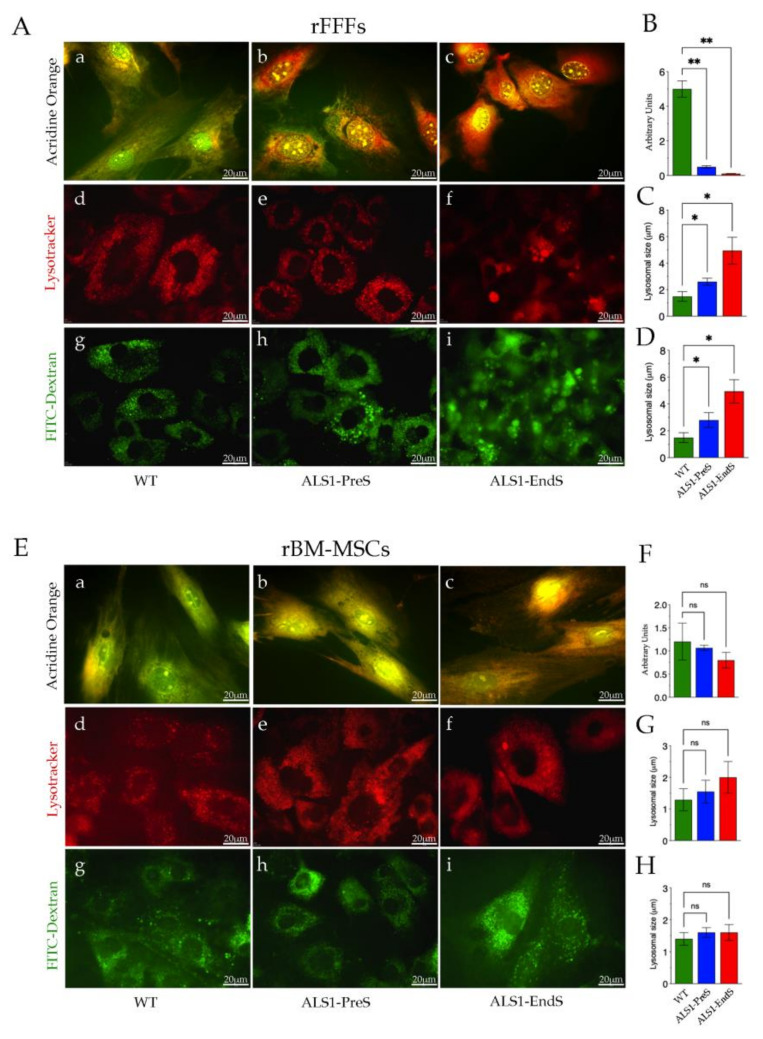
Lysosomes homeostasis and size in rFFFs (**A**–**D**) and rBM-MSCs (**E**–**H**) from ALS1 and WT rats. (**A**,**a**–**c**;**E**,**a**–**c**) Representative images of Acridine Orange staining. Scale bar = 20 μm. (**B**,**F**) Quantification of the staining of Acridine Orange (see Method Section 2.11 and Figure S5 in Supplementary File for details). (**A**,**d**–**f**;**E**,**d**–**f**) Representative images of Lysotracker staining (Scale bar = 20 μm.) and (**C**,**G**) related quantification (see Section 2.11 for details). (**A**,**g**–**i**; **E**,**g**–**i**) Representative images of FITC-Dextran staining (Scale bar = 20 μm), and (**D**,**H**) the quantification plot (see Method Section 2.11 and Figure S5 in Supplementary File for details). All reported results are the mean ± SD of three independent experiments. ns, not significant, * *p* < 0.05, ** *p* < 0.01.

To further investigate the lysosomal compartment dysfunction, we analyzed the lysosomes homeostasis and size, in rFFFs (Figure 7A–D) and rBM-MSCs (Figure 7E–H) ALS1 in vitro models.

Lysosome homeostasis was evaluated by using the lysotropic dye Acridine Orange, which, in a pH-dependent manner, stains the nucleic acids in the nucleus and cytoplasm in green, and the acidic compartments, such as lysosomes and autophagolysosomes, in orange/red [43,44] (Figure 7). After the treatment, WT rFFFs follow the canonical pH gradient staining with a large green area/small orange spots, whereas the scenario was worsening in ALS1-fibroblasts, with the prevalence of large orange/red staining, that increased with the progression of the disease (Figure 7A(a–c),B). The phenomenon was only slightly observable in ALS1 rBM-MSCs since the little increase of orange/red spots in diseased cells was not significant compared to the canonical acridine orange staining in WT stem cells (Figure 7E(a–c),F). The quantitative analysis of the ratio green/red signal also indicated the presence of metabolic alterations in ALS1-rFFFs rather than the ALS1-rBM-MSCs (Figure 7B,F). 

The lysosomes size was evaluated by using the lysosomal tracker (Lysotracker), and the fluorescent molecule FITC-Dextran (Figure 7A,E) [45]. Lysosomes’ size increased with the onset of the disease since the organelles were bigger in rFFFs ALS1-EndS with respect to ALS1-PreS that were higher than lysosomes in WT cells (Figure 7A(d–f),C). No significant differences were found in the lysosomal size in ALS1-PreS, ALS1-EndS, and WT rBM-MSCs (Figure 7E(d–f),G). 

Comparable results were obtained in cells treated with FITC-Dextran. We observed larger lysosomes in both types of ALS1 fibroblasts (order ALS-EndS>ALS-PreS) with respect to WT counterparts (Figure 7A(g–i),D), whereases, no significant variations were observed in stem cells from ALS1 and WT (Figure 7E(g–i),H). 

Thus, Lysotracker and FITC-Dextran treatments revealed a significant difference in the size of lysosomes in ALS1-rFFFs compared to ALS1-rBM-MSCs but also indicated a similar lysosomal distribution area in both ALS1-cell types and the WT counterparts (Figure 7A,E).

### 3.5. SOD1* Is Stored in the Lysosomes of ALS1-rFFFs and ALS1-rBM-MSCs In Vitro Models

**Figure 8:**
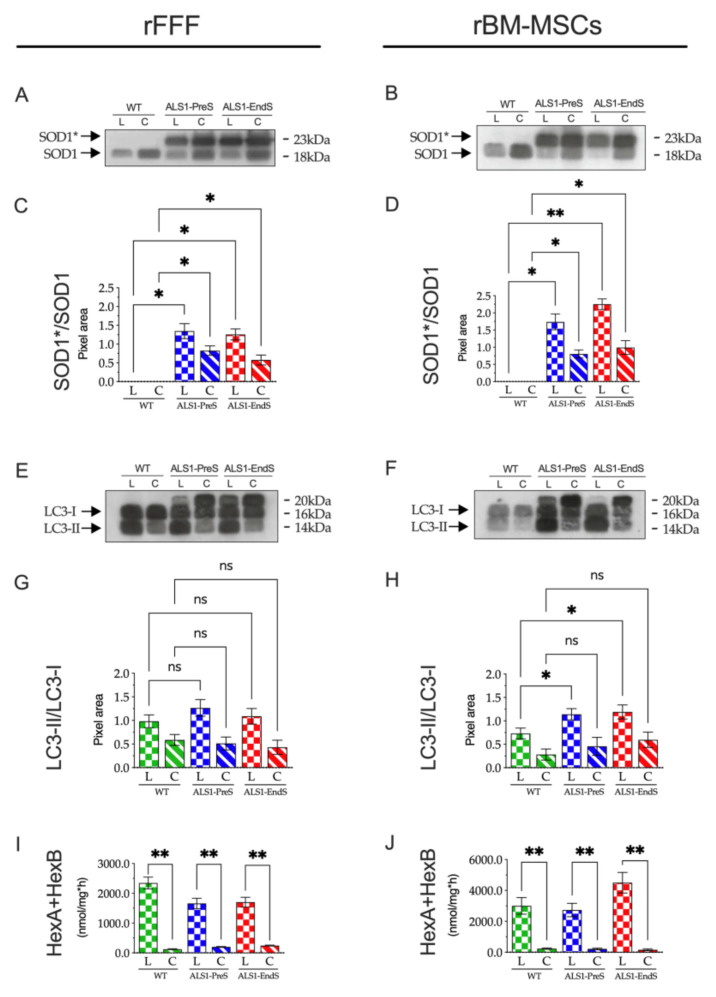
Expression of SOD1* and LC3 in lysosomal and cytoplasmatic fractions of rFFFs and rBM-MSCs from ALS1-PreS and ALS1-EndS animal models. (**A**,**B**) Representative Western blotting analysis is expressed as a ratio of the intensity of the SOD1*/SOD1 bands. (**C**,**D**) Densitometric analysis is expressed as a ratio of the intensity of the SOD1*/SOD1 bands. Results are reported as the mean± SD of three replicates for each sample. * *p* < 0.05, ** *p* < 0.01. (**E**,**F**) Representative Western blotting analysis of LC3-I and LC3-II in ALS1 and WT cell fractions. (**G,H**) Densitometric analysis is expressed as a ratio of the intensity of the LC3-II/LC3-I bands. Results are reported as the mean ± SD of three replicates for each sample. * *p* < 0.05, ** *p* < 0.01. (**I**,**J**) Activity of Hexosaminidase (HexA + HexB) in ALS1 and WT cell fractions. Enzyme activity was measured by using the specific fluorogenic substrate MUG (see Section 2.14 for details). Results were expressed as the mean ± SD of three independent experiments, each in triplicates. L, lysosomal fraction; C, cytoplasmatic fraction. ns, not significant, * *p* < 0.05, ** *p* < 0.01.

To correlate the lysosomal dysfunction with the SOD1* storage, we analyzed the presence of SOD1* in the lysosomes of both diseased and WT rFFFs and rBM-MSCs (Figure 8A–D).

After the subcellar fractionation, the lysosomal (L) and the cytoplasmatic (C) fractions of each cell type were analyzed by Western blotting. Results showed a comparable SOD1 pattern in the L and C fractions of both WT rFFFs and rBM-MSCs, consisting of one band corresponding to the naïve SOD1 (Figure 8A,B) and two bands corresponding to naïve SOD1 and mutated SOD1* forms in Pre-symptomatic and End-stage rFFFs and rBM-MSCs (Figure 8A,B). The densitometric analysis confirmed the higher expression of mutant SOD1* compared to the SOD1 band in the L fractions and indicated that the enzyme is stored in the lysosomes of ALS1-PreS and ALS1-EndS rFFFs and rBM-MSCs (Figure 8C,D).

Finally, we wanted to evaluate the contribution of autophagy to the observed lysosomal dysfunction in rFFFs and rBM-MSCs from ALS1-PreS and ALS1-EndS animal models.

Interestingly, the analysis of the expression of LC3, a marker of active autophagy, in the above subcellular fractions from ALS1 and WT cells revealed a different scenario in rFFFs and rBM-MSCs (Figure 8E–H). In both fibroblasts and stem cells of WT, ALS-PreS, and ALS1-EndS we found the LC3-I and its cleaved form LC3-II, with the LC3-II higher in the L compared to the C fraction (Figure 8E,F), thus indicating an active autophagic process (Figure 8E,F). However, whether in rFFFs the ratio of LC3-II/LC3-I was comparable between the WT and ALS1 counterparts (Figure 8E,G), the ratio LC3-II/LC3-I increased with the progression of the disease in rBM-MSCs (Figure 8F,H), suggesting that the process was more pronounced in stem cells. Interestingly, both ALS1 cell types expressed the LC3 20kD band which was absent in WT cells, but already described in cancer cells [46].

The highest activity of the lysosomal Hexosaminidase (HexA + HexB) activity (Figure 8I,J) and the expression of Lamp1, a protein of the lysosomal membrane (Figure S6, Supplementary File) confirmed the correct lysosomal fraction separation in both cell types.

These results demonstrated the presence of SOD1* storage in lysosomes of both ALS1 non-neural cells but also highlighted two different autophagic cellular responses that, together with the above results, suggest a potential metabolic safety mechanism for ALS1 stem cells rather than ALS1 rFFFs.

## 4. Discussion

In this work, we demonstrated the involvement of the lysosomes’ dysfunction in the progression of ALS1 disease through an integrated study performed in different tissues and non-neuronal cells isolated from the ALS1 transgenic rat^G93A^ model. We documented that the storage of human mutant SOD1* in the lysosomes of primary fibroblasts and bone marrow-mesenchymal stem cells from rat^G93A^ is associated with the alteration of glycohydrolases and proteases levels in both cell types, but, only in ALS1-rFFFs the lysosomes lost homeostasis and increased drastically in size, whereas in ALS1-rBM-MSCs the neglectable metabolic dysfunction, make these cells comparable to WT counterparts. We addressed this phenomenon to a safety mechanism perhaps associated with an enhanced lysosomal autophagic activity in ALS1-rBM-MSCs that, might balance the impact of the formation of SOD1* aggregates, even in stem cells at the terminal stage of the disease.

Up to now, the molecular pathways leading to ALS pathophysiology are almost unclear. Therefore, the effort has been made to develop different types of animal models that recapitulate the diverse forms of ALS [7]. In this regard, both transgenic mice and rats, that express the human *SOD1* gene carrying the mutation SOD1^G93A^, recapitulate the feature of ALS1 [23], offering a tool to explore the development of therapeutic strategies for the cure of the disease, and are an appropriate source of ALS1-cells that could be explored for elucidating the pathological molecular pathways. 

Compared to the published studies mainly conducted in ALS1 neural cells (e.g., motor neurons, astrocytes [47,48,49,50,51,52,53]) we chose primary fibroblasts (rFFFs) and bone marrow- mesenchymal stem cells (rBM-MSCs) from the ALS1 rat^G93A^ as cell models. We demonstrated that the presence of the hallmarks of the disease broad in non-neural cells, and the signs increase with the progression of the disease. Therefore, both cell types may be suitable in vitro systems to explore the molecular pathophysiology of ALS1. 

Both rFFFs and rBM-MSCs overexpressed SOD1*, which increased with the progression of the disease (ALS1-EndS >ALS-PreS). As in the brain, muscle, and other tissues collected from ALS1-PreS and ALS1-EndS at sacrifice, in both cell types, we found a similar pattern of expression consisting of the naïve SOD1 and the human mutant SOD1* that was absent in related WT counterparts. rFFFs and rBM-MSCs from ALS1-EndS rat^G93A^ also showed large acidophilic aggregates absent in ALS1-PreS and WT systems. Moreover, both ALS1-cells have an increased proliferation rate rather than their WT counterparts. Up to now, we do not have a clear explanation of this phenomenon, however, the correlation of the overexpression of naive SOD1 has been documented in the non-small cell lung cancer where the rate of proliferation was associated with the activity of miR-409-3p/SOD1/SETDB1 pathways [54]. Alteration of the proliferation of skeletal muscle satellite cells isolated from mouse SOD1-G93A was also described [55]. These evidences suggest a potential involvement of the SOD1 in the proliferation process. We speculate that this phenomenon might be correlated with an abnormal presence of SOD1 protein in the nucleus of both ALS1 cell models. In this regard, confirming previous reports [13,14], we found a reduction of SOD1 expression in the nuclei of both ALS1-rFFFs and ALS1-rBM-MSCs (Figure 3G,H), thus further validating our non-neural cell model.

Hence, we used rFFFs and rBM-MSCs as suitable in vitro non-neural cell models to study the contribution of the lysosomal dysfunction to the ALS1 progression.

Lysosomes are organelles specialized in the degradation of macromolecules (through the activity of more than 50 acid hydrolases) and the recycling of basic molecular components into biosynthetic and bioenergetics materials for maintaining the cell homeostasis, thus functioning as a hub for the integration of cellular signals [56,57]. The autophagy, a tightly regulated degradation lysosome-dependent process, concurs with this biological process [56,57]. Therefore, deregulation of molecular pathways leading to lysosomal functions may steer the cell status from a physiological to a pathological condition and highlighted that the maintenance of the lysosome homeostasis serves as a safety mechanism for keeping the health status of cells and tissues [56,57].

The potential involvement of the lysosomal disturbances in ALS has been highly explored in the last years [4,58,59,60,61], due to some evidence showing impaired lysosome functionality, and the dysfunction of the associate autophagic process in ALS post- mortem tissues [62,63]. For instance, mutations of the *C9orf72* (Chromosome 9 open reading frame 72) gene have been shown to affect the activity of Rab protein, with the induction of endo-lysosomal pathway dysfunction, altered autophagy, and accumulation of protein aggregates in ALS-FTD1 [62]. Mutations in the genes *UBQLN2* (ubiquilin-2), *VCP* (valosin-containing protein), *OPTN* (optineurin), *TBK1* (serine/threonine-protein kinase-TBK1), respectively cause of ALS15, ALS14, ALS12, ALS-FTD4, are also associated with the lysosomes and autophagy impairment [64]. Moreover, lysosomal dysfunction, or/and the associated autophagic pathways, are also described in the other ALS forms [4,58,59,60,61], however, the precise effect in the pathogenesis of ALS is still controversial and is under extensive exploration. 

In this regard, our results confirmed the lysosomal dysfunction in ALS1 tissues, rFFFs, and rBM-MSCs and added new findings on the impaired metabolic pathways.

First, our work offered new evidence of the aberrant levels of a plethora of lysosomal enzymes in non-neural cells and tissues as in the brain of ALS1 compared to the WT system.

All glycohydrolases (Hex, Glu, Man, Gal, GALC) and proteases (CatB, CatD, CatS) levels were altered in ALS1 samples compared to the related WT counterparts. Of note, although those enzyme alterations levels were in a wide range, the highest alterations were found in tissues and non-neural cells from ALS1-EndS rat^G93A^, and in general, the abnormal expression increased with the progression of the disease. 

Compared to the study of Dodge and co-authors [65], performed investigating some of the lysosomal glycohydrolases that we tested (Hex, Gal, GALC) in the spinal cord from the ALS1 mouse^G93A^, we confirmed the high increase of Hex (both HexA + HexB and HexA) and Glu in the ALS1-EndS whole brain, where also we found increased the activity of Man, whereas, the other enzyme Gal and GALC were drastically reduced. Additionally, we documented alteration in muscle (the increase of HexA + HexB, HexA, Glu, Man, Gal; the decrease of GALC), heart (the reduction of HexA + HexB, HexA, Glu, Man, Gal, GALC), rFFFs (the decrease of HexA + HexB, HexA, Glu, Man, Gal, GALC), and rBM-MSCs (the reduction of HexA + HexB, HexA, Glu, Man; the decrease of Gal, GALC). 

The overall results agreed with the reported observation showing the dysregulation of the glycosphingolipids metabolism in ALS1 mouse^G93A^ and ALS1 mouse SOD1^G86A^ [6,65]. It was suggested that the altered levels of these enzyme activities affect the canonical catabolism of related substrates (e.g., Hex: GM2 Ganglioside; Glu: Glucosylceramide; Gal: GM1 Ganglioside; GALC: Galactosylceramide) and dysregulate the consequent pathways that contributed to the ALS1 pathogenesis. These findings are supported by a recent publication reporting that a monogenic form of ALS is caused by SPTLC1 variants that disrupt the normal homeostatic regulation of serine palmitoyltransferase (SPT) by ORMDL sphingolipid biosynthesis regulator proteins, with the consequent upregulation of SPT activity and increased levels of glycolipids [66].

Interestingly, among the glycohydrolases analyzed, the GALC enzyme was also decreased in muscle, heart, rFFFs, and rBM-MSCs at the terminal stage of the disease (ALS1-EndS). Yet, these results support the above correlation with the alteration of glycosphingolipids metabolism, and with the accumulation of undegraded galactocerebroside, which is the first cause of Krabbe Disease, a genetic lysosomal storage disorder [67,68]. Indeed, alteration of GALC activity was also described by our group in the plasma of patients affected by Mild Cognitive Impairment compared to Alzheimer’s disease patients [34,38,41]. These results were supported by other studies showing alteration of the above-mentioned glycohydrolases in other neurodegenerative diseases, although in a wide range, and shed light on the tight biological role of the lysosome in neurodegenerative diseases progression (AD, PD, HD), thus resembling the LSDs [69,70,71,72,73].

To the best of our knowledge, data exploring the expression of CatB, CatD, and CatS in ALS are quite a few, however reduction of CatB (25 KDa active form) has been described in the ALS1 motor neuron [74]. Similarly, altered levels of CatD were reported to be involved in the ALS1 motor neuron degeneration, whereas, CatS was found up-regulated in the spinal cord and some brain regions in a model of ALS1 [75].

Our results confirmed the above-described downregulation of CatB, CatD, and CatS in the brain of rat^G93A^ at the End-Stage of the disease. This trend was opposite in the ALS1-EndS muscle, while the expression levels of these proteases in the heart, and non-neural cells were varied. 

Collectively, these results spotlighted the correspondence of altered levels of glycohydrolases and proteases with the progression of the disease in the different ALS1-tissues and non-neural cells. Furthermore, they might help to understand the absence of the degradation of misfolded SOD1* aggregates in the lysosomes and the consequent formation of SOD1* storage.

In this regard, the ALS1-non-neural cell models allowed us to deeply explore the correlation between lysosomal dysfunction and ALS1 disease progression. We found an utmost dysregulation of the homeostasis of lysosomes in ALS1-rFFFs, also extended to the whole cells, compared to WT cells. Of note, the metabolic alterations (assessed by acridine orange staining and Hematoxylin/Eosin staining) increased from PreS-rFFFs to EndS-rFFFs and was supported by the high increase of the size of lysosomes (both Lysotracker and FITC-Dextran staining) and foremost by the presence of SOD1* in the lysosomes of diseased cells and the absence in WT counterparts.

Conversely, although we demonstrated the presence of SOD1* in the lysosomes of ALS1-rBM-MSCs compared to WT, and the presence of acidophilic aggregates in the ALS1-EndS stem cells, no significant metabolic alterations or change of lysosomes’ size were monitored in both ALS1-PreS and ALS1-EndS rBM-MSCs.

The different expression of LC3-II in the lysosomal fraction of both ALS1-rFFFs and ALS1-rBM-MSCs in part clarify the phenomenon. Despite more experiments are necessary to elucidate these molecular events, the higher expression of LC3-II in the lysosomal fraction of ALS1-rBM-MSCs compared to those of WT-rBM-MSCs, and its similar expression in the lysosomal fraction of ALS1- and WT-rFFFs, indicated an increase of the autophagic activity in ALS1-rBM-MSCs compared to ALS1-rFFFs. Therefore, collecting these results, we suggest that the increase of the autophagic activity in stem cells might preserve the clearance of the lysosomes and the cellular status compared to fibroblasts that were significantly compromised.

Currently, the relation between ALS1 and autophagy is a debated issue due to contrasting evidence either showing the association of impaired autophagy and motor neuron degeneration or that the autophagic clearance of mutant SOD1 is beneficial to ALS1 [2,3,4,76,77]. In this regard, our results, highlighting the absence of metabolic alteration in ALS1-rBM-MSCs, support the latter evidence, although we are aware that the phenomenon must be extensively investigated. 

## 5. Conclusions

In conclusion, to the best of our knowledge, our results are pioneering on demonstrating the alteration of a plethora of lysosomal enzyme activity, glycohydrolases, and proteases, and the SOD1* storage in both rFFFs and rBM-MSCs non-neural cells from the model of ALS1, the rat^G93A^. These lysosomal enzymes alterations increased with the progression of the disease, thus, supporting other studies, on the potential involvement of lysosome dysfunctions as the events that could be associated with ALS pathogenesis.

Interestingly, our cell models shed light on the complexity of ALS1 and lysosome alterations, showing diverse cell responses to the ALS1 pathology. While in both cell types, the lysosomal dysfunction correlated with storage of SOD1*, only in ALS1-rFFFs, these events agreed with abnormal cell homeostasis. We hypothesized that the neglectable metabolic changes observed in ALS1-rBM-MSCs could be a consequence of peculiar regulation of the lysosomal-dependent autophagy activity that could activate a safety mechanism that restored the metabolic alteration in ALS1 stem cells.

Despite more experiments needed to investigate better this phenomenon, the overall results support the possibility of intervening on ALS1 pathology through the modulation of this fascinating critical cellular mechanism. 

## Supplementary Materials

The following are available online at https://www.mdpi.com/article/10.3390/biomedicines9091080/s1, Figure S1: Human mutant SOD1* is overexpressed in tissues-derived from ALS1 rat^G93A^ models and WT, Figure S2: Expression of Type I and Type III Collagen in ALS-PreS, ALS-EndS, and WT rFFFs, Figure S3: Osteogenic and adipogenic differentiation of rBM-MSCs from ALS-PreS, ALS-EndS, and WT animal model, Figure S4: Western blotting of CatD, CatS, and CatB in ALS1-tissues, -rFFFs, and -rBM-MSCs, Figure S5: Schematic of Lysosomes quantification workflow, Figure S6: Expression of LAMP1 in the Lysosomal (L) and Cytoplasmatic (C) fractions of rFFFs and rBM-MSCs from ALS1-PreS and ALS1-EndS animal models.
