# Supplementary material for: Storage of Mutant Human SOD1 in Non-Neural Cells from the Type-1 Amyotrophic Lateral Sclerosis ratG93A Model Correlated with the Lysosomes’ Dysfunction
Source: Biomedicines. 2021 Aug 24;9(9):1080. doi: 10.3390/biomedicines9091080 (PMC8470315; doi:10.3390/biomedicines9091080)
Supplement: Supplementary file 1 [file biomedicines-09-01080-s001.zip › biomedicines-1309193-supplementary.pdf]

Article

Supplementary Files

Storage of mutant SOD1 in non-neural cells from type-1 of Amyotrophic Lateral Sclerosis rat^{G93A} model correlated with the Lysosomes' dysfunction.

Ilaria Bicchi[1,2*], Francesco Morena[1*], Chiara Argentati[1], Laura Rota Nodari[3], Carla Emiliani[1], Maurizio Gelati[2], Angelo L. Vescovi[2], Sabata Martino[1§]

[1] Department of Chemistry, Biology and Biotechnology, University of Perugia, Via del Giochetto, 06123 Perugia, Italy; ilabi1983@gmail.com (I.B.) ;francesco.morena@unipg.it (F.M.); chiara.argentati@unipg.it (C.A); carla.emiliani@unipg.it (C.E); sabata.martino@unipg.it (S.M.).
[2] IRCCS Casa Sollievo della Sofferenza, Viale Cappuccini 1, 71013 San Giovanni Rotondo, Foggia, Italy; m.gelati@css-medel.it (M.G.) vescovia@gmail.com (A.L.V.)
[3] Department of Biotechnologies and Biosciences, University of Milano Bicocca, Piazza della Scienza 2, 20126 Milan, Italy; laura.rotanodari@gmail.com (L.R.N.)
§ Correspondence: Sabata Martino; sabata.martino@unipg.it (S.M.). Tel.: (+39 0755857442)

*I.B. and F.M. did equal contribution to this work, and therefore are co-first authors.

Citation: Lastname, F.; Lastname, F.; Lastname, F. Title. *Biomedicines* **2021**, *9*, x. https://doi.org/10.3390/xxxxx

Academic Editor: Firstname Lastname

Received: date
Accepted: date
Published: date

Publisher's Note: MDPI stays neutral with regard to jurisdictional claims in published maps and institutional affiliations.

Copyright: © 2021 by the authors. Submitted for possible open access publication under the terms and conditions of the Creative Commons Attribution (CC BY) license (https://creativecommons.org/licenses/by/4.0/).

Content:

Supplementary Figure S1A-S1B

Supplementary S2

Supplementary Figure S3A-S3B

Supplementary Figure S4A-S4C

Supplementary Figure S5

Supplementary Figure S6A-S6D

Figure S1

Human mutant SOD1* is overexpressed in tissues-derived from ALS1 rat^{G93A} models and WT.

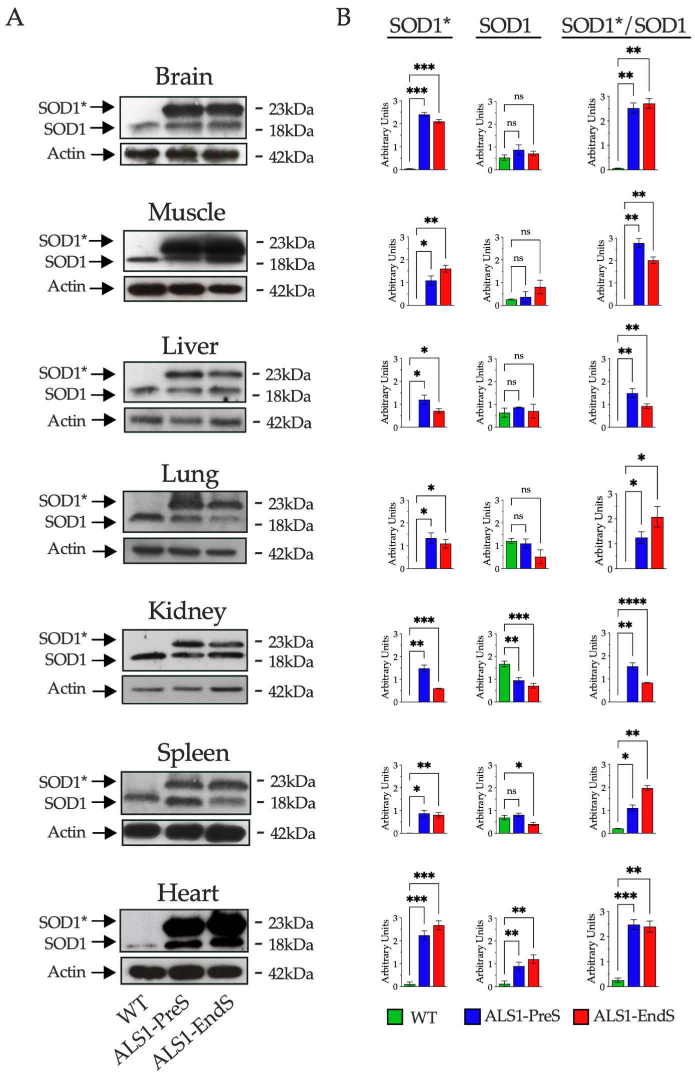

Figure S1. A) Representative Western blotting analysis of the expression of naïve SOD1 and human mutant SOD1* in several tissues from ALS1-PreS, ALS1-EndS and WT rats. B) Densitometric analysis of the SOD1*, SOD1 and SOD1*/SOD1 ratio. Results are reported as mean ± SD of three independent experiments. * $p < 0.05$, ** $p < 0.01$, *** $p < 0.001$. Details are reported in the method and results sections in the main text.

Figure S2

Expression of Type I and Type III Collagen in ALS1-PreS, ALS1-EndS, and WT rFFFs.

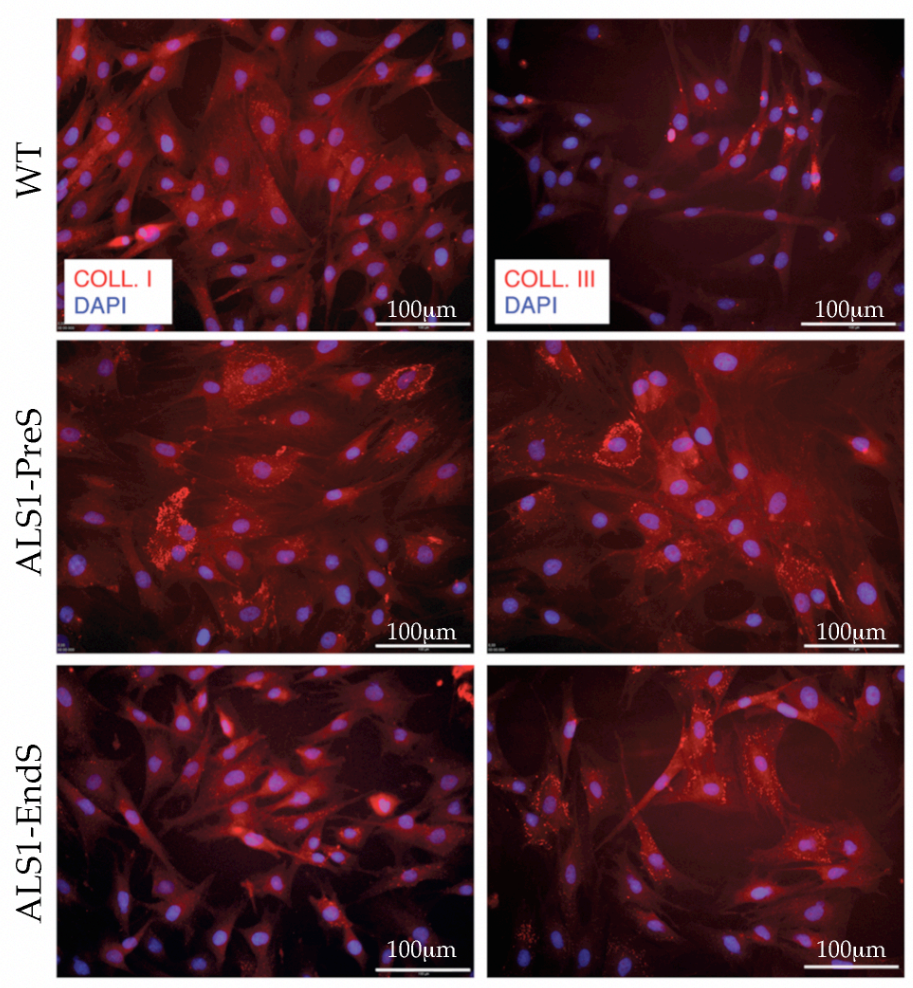

Representative immunofluorescences of **Type I and Type III Collagen** (anti- Type I and anti-Type III Collagen; red), Nuclei (4′,6-diamidino-2-phenylindole, DAPI, blue).

Figure S3

Osteogenic and adipogenic differentiation of rBM-MSCs from ALS1-PreS, ALS1-EndS, and WT animal model.

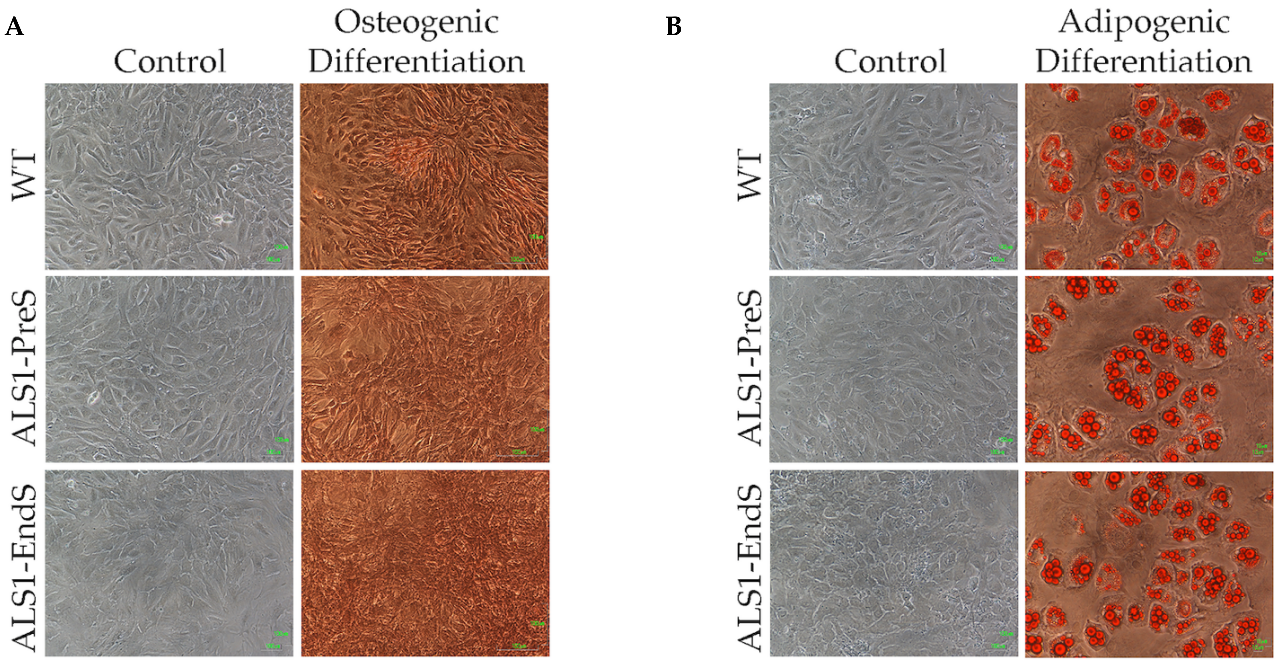

Representative images of osteogenic and adipogenic differentiation of rBM-MSCs from ALS1-PreS, ALS1-EndS, and WT animal model.

A) Osteogenic differentiation - Alizarin Red staining showed the presence of calcium deposits in differentiated stem cells that were absent in control undifferentiated counterparts. No differences were evidenced between diseased and healthy control stem cells.

B) Adipogenic differentiation – OIL Red staining revealed the presence of lipid drops in deposits in differentiated stem cells that were absent in control undifferentiated counterparts. No differences were observed between diseased and healthy control stem cells.

Figure S4

Western blotting of CatD, CatS, and CatB in ALS1-tissues, -rFFFs, and -rBM-MSCs

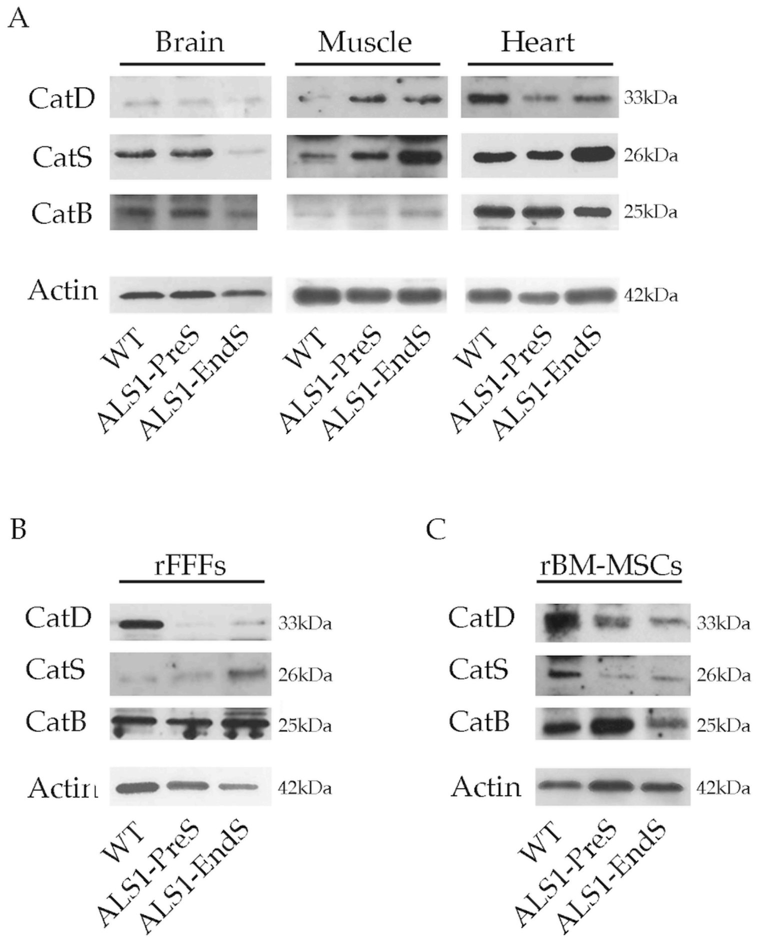

Representative Western blotting of CatD, CatS, and CatB in tissues (A), rFFFs (B), and rBM-MSCs (C). See Figure 5, 6A,6B in the Results section and Method section in the main text for details.

Figure S5

Schematic of Lysosomes quantification workflow

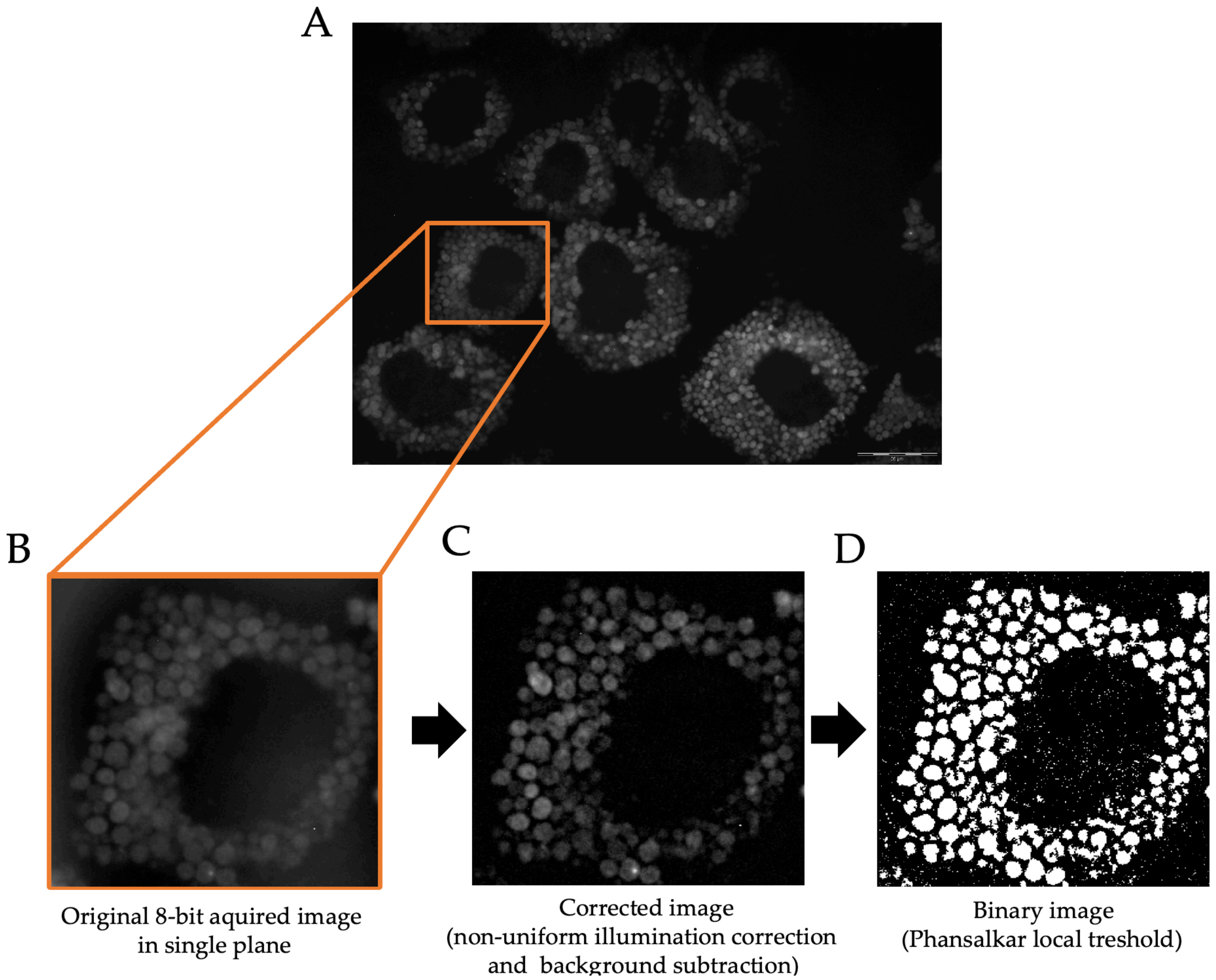

Representative workflow for quantification of lysosomes size. For each fluorescence image (A), stained cells were evaluated (B). Both Lysotracker and FITC-Dextran allow us to mark specifically the lysosomes. Before quantification, images were pre-processed (C; see method section 2.11 main text) and where threshold with "Auto Local threshold"-> "Phansalkar" (D). The binary images were used for lysosomes size measurement in FIJI with the "Analyze Particles" tools and "Size (pixel^2)" parameter set to "50-Infinity.

Figure S6

Expression of LAMP1 in the Lysosomal (L) and Cytoplasmatic (C) fractions of rFFFs and rBM-MSCs from ALS1-PreS and ALS1-EndS animal models

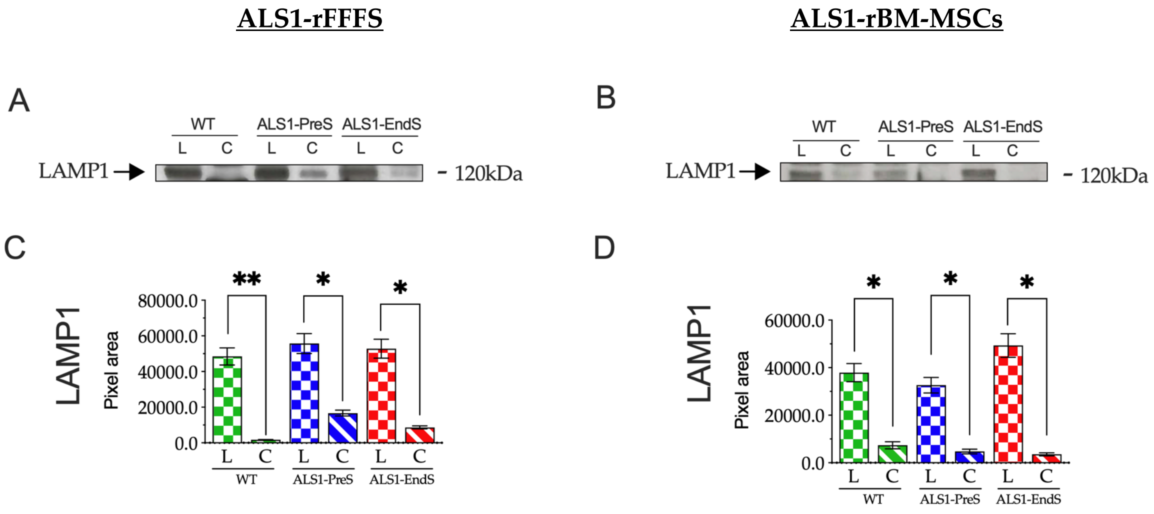

Expression of LAMP1 (ABCD) in subcellular fractions of rFFFs and rBM-MSCs from ALS1-PreS and ALS1-EndS animal models. Representative Western blotting analysis of LAMP1 in ALS1 and WT cell fractions. C,D) Densitometric analysis of LAMP1. Results are reported as the mean± SD of three replicates for each sample. *$p < 0.05$, **$p < 0.01$. L, lysosomal fraction; C, cytoplasmatic fraction. See Section 2 in the main text for details.
